# Vancomycin-releasing cross-linked collagen sponges as wound dressings

**DOI:** 10.17305/bjbms.2019.4496

**Published:** 2021-02

**Authors:** Jan Miroslav Hartinger, Peter Lukáč, Petr Mitáš, Mikuláš Mlček, Michaela Popková, Tomáš Suchý, Monika Šupová, Jan Závora, Václava Adámková, Hana Benáková, Ondřej Slanař, Martin Šíma, Martin Bartoš, Hynek Chlup, Tomáš Grus

**Affiliations:** 1 Department of Clinical Pharmacology and Pharmacy, Institute of Pharmacology, First Faculty of Medicine, Charles University and General University Hospital in Prague, Prague, Czech Republic; 2 2^nd^ Department of Cardiovascular Surgery, First Faculty of Medicine, Charles University and General University Hospital in Prague, Prague, Czech Republic; 3 Institute of Physiology, First Faculty of Medicine, Charles University and General University Hospital in Prague, Prague, Czech Republic; 4 Department of Composites and Carbon Materials, Institute of Rock Structure and Mechanics, Academy of Sciences of the Czech Republic, Prague, Czech Republic; 5 Department of Mechanics, Biomechanics and Mechatronics, Faculty of Mechanical Engineering, Czech Technical University in Prague, Prague, Czech Republic; 6 Institute of Medical Biochemistry and Laboratory Diagnostics, First Faculty of Medicine, Charles University and General University Hospital in Prague, Prague, Czech Republic; 7 Department of Stomatology, First Faculty of Medicine, Charles University and General University Hospital in Prague, Prague, Czech Republic

**Keywords:** Wound dressing, sponge, carp, freshwater fish, collagen, vancomycin, infection, crosslinking, MRSA

## Abstract

The study presents a novel vancomycin-releasing collagen wound dressing derived from *Cyprinus carpio* collagen type I cross-linked with carbodiimide which retarded the degradation rate and increased the stability of the sponge. Following lyophilization, the dressings were subjected to gamma sterilization. The structure was evaluated via scanning electron microscopy images, micro-computed tomography, and infrared spectrometry. The structural stability and vancomycin release properties were evaluated in phosphate buffered saline. Microbiological testing and a rat model of a wound infected with methicillin-resistant *Staphylococcus aureus* (MRSA) were then employed to test the efficacy of the treatment of the infected wound. Following an initial mass loss due to the release of vancomycin, the sponges remained stable. After 7 days of exposure in phosphate buffered saline (37°C), 60% of the material remained with a preserved collagen secondary structure together with a high degree of open porosity (over 80%). The analysis of the release of vancomycin revealed homogeneous distribution of the antibiotic both across and between the sponges. The release of vancomycin was retarded as proved by *in vitro* testing and further confirmed by the animal model from which measurable concentrations were observed in blood samples 24 hours after the subcutaneous implantation of the sponge, which was more than observed following intraperitoneal administration. The sponge was also highly effective in terms of reducing the number of colony-forming units in biopsies extracted from the infected wounds 4 days following the inoculation of the wounds with the MRSA solution. The presented sponges have ideal properties to serve as wound dressing for prevention of surgical site infection or treatment of already infected wounds.

## INTRODUCTION

To date, the application of vancomycin-releasing collagen sponges as wound dressings has not been studied to any significant extent. Current approaches involve either the incorporation of other antibiotics into collagen sponges [1-3] or the use of vancomycin as one element of hydroxyapatite beads in the treatment of musculoskeletal injuries [4,5]. Moreover, various studies have described the administration of a vancomycin powder directly on surgical wounds so as to prevent wound infection following spinal, cardiac, or vascular surgery, with good effects [6,7]. In addition to the prevention of infection, a hemostatic effect is desirable in the postoperative wound healing process. Vander Salm et al. mixed a vancomycin powder with thrombin and an absorbable gelatin paste, which served for hemostatic purposes [8]. Further, Hamman et al. described mixing of vancomycin with platelet-rich plasma and calcium-thrombin [7]. It is proposed that a vancomycin-releasing hemostatic collagen sponge would be ideal for this purpose since it consists of a ready-to-use device that does not require preparation during surgery via the mixing of the antibiotic with hemostatic compounds.

Since neither vancomycin nor aminoglycosides do not readily penetrate into tissues [9], their effect in terms of postoperative wound treatment and prophylaxis may be insufficient in the case of systemic administration, and systemic toxicity may follow; therefore, local administration is preferable. High local vancomycin levels were achieved with no signs of nephrotoxicity [10], when vancomycin-impregnated bone grafts in combination with an aminoglycoside-containing cement were used in hip joint replacement surgery [10]. Following the application of 1000 mg of vancomycin powder during surgery for spinal deformity in children, supratherapeutic levels were achieved in a fluid drained by a subfascial drain up to the second postoperative day (403 mg/L, 251 mg/L, and 115 mg/L for day 0, day 1, and day 2, respectively) and the same study recorded non-toxic levels in plasma (2.5 mg/L, 1.9 mg/L, and 1.1 mg/L for day 0, day 1, and day 2, respectively) [11].

Mammal collagens, such as calf or bovine, are the most used collagen types for biomaterial engineering [4,5].

However, the use of mammal collagen may be complicated by an allergic reaction induced in 3–4% of the population [12]. Therefore, fish collagen is being subjected to intensive research with an emphasis on its application for medical purposes [3,13-16]. Since fish-derived collagen exhibits a lower melting temperature and less reproducible properties than mammal collagen [17], it must be crosslinked in order to obtain a product that exhibits reproducible properties [14]. A further reason for crosslinking is to enhance durability during the sterilization process since non-crosslinked collagen is significantly degraded upon exposure to gamma rays [18]. Therefore, if gamma sterilization is a part of the fabrication of the ready-to-use medical device, crosslinking provides a reliable way to preserve the properties of both fish and mammal collagen [3,19]. One of our previous studies showed successful testing of a gentamicin-releasing carbodiimide cross-linked with freshwater fish (*Cyprinus carpio, C. carpio*) collagen wound dressing on a rat model of surgical site infection [3]. The current study presents the results of structural, drug-releasing, and microbiological testing of an animal model of infected wound treated with a novel vancomycin-releasing cross-linked freshwater fish-derived collagen wound dressing.

## MATERIALS AND METHODS

### Collagen extraction

Collagen type I was isolated from freshwater fish skin (*C. carpio*, *Třeboň carp*, Třeboň fishery Třeboň, Czech Republic) by adapting the procedure published by Bell et al. [20]; all the fish were of food grade and controlled breeding. The degreasing procedure was performed in 70% ethanol (Penta, Czech Republic), followed by washing 30 min in distilled water. Collagen was extracted by 0.001 vol% acetic acid (Penta, Czech Republic) for 48 h under continuous stirring, followed by centrifugation at 10,000 r/min for 1 h. The supernatant was precipitated in 0.1 M NaOH (Penta, Czech Republic) at a ratio of 6:1 (v/v) to neutralize acetic acid. The mixture was then re-centrifuged at 10,000 r. min^-1^ (50 min). The collagen precipitates were again solubilized in 0.001 vol% acetic acid, frozen at -15°C and lyophilized (BenchTop 4KZL, VirTis, Prague, Czech Republic). The purity of the isolated collagen was verified repeatedly by a number of methods, e.g., the determination of the lipid content and inorganic impurities. Moreover, the collagen structure was verified through electrophoresis and by infrared spectrometry. Possible cytotoxicity of isolated collagen was determined by *in vitro* tests; detailed results have been published elsewhere [21].

### Preparation of collagen sponges

Sponges were prepared from a collagen dispersion (0.8 wt%) in deionized water; after swelled collagen (8°C, 1 h) was homogenized by disintegrator (10,000 rpm, 10 min and 10,000 rpm, 2 min with 20 min delay at 20°C). The resulting product was frozen at -80°C for 5 h and lyophilized.

Collagen sponges were crosslinked in a 95% ethanol solution containing N-(3-dimethylaminopropyl)-N-ethylcarbodiimide hydrochloride (EDC) and N-hydroxysuccinimide (NHS) at a weight ratio of 4:1; a 3-h reaction period at 37°C with EDC and NHS (Sigma-Aldrich, Steinheim, Germany) was followed by washing with 0.1 M Na_2_HPO_4_ (2 × 45 min), rinsing in deionized water for 30 min, freezing at -15°C (5 h), and lyophilization.

Each of the cross-linked sponges with dimensions of approximately 40 mm × 40 mm was impregnated with 5 mL of 95% wt ethanol solution with 64 mg of vancomycin (Mylan S.A.S, France). After impregnation, sponges were frozen and lyophilized. Finally, specimens were cut to the dimensions suitable for further analyses. The final collagen to the vancomycin weight ratio was 5:4.

The sterilization of vancomycin-loaded sponges was performed by gamma sterilization. All samples were packed and exposed to a nominal dose of 25 kGy (BIOSTER, a.s., Veverská Bítýška, Czech Republic). The samples used in all experiments were sterilized.

### Microstructure of collagen sponges

#### Scanning electron microscopy (SEM)

The microstructure of sponges was characterized by SEM (Quanta 450 Microscope, FEI, Hillsboro, Oregon, USA) in high vacuum mode. Prior to microscopy, the cross-sections of sponges were covered by a thin layer of gold (Emitech K550X ion Sputter, Quorum Technologies, United Kingdom).

#### Micro-computed tomography (CT)

The quantification of structural parameters was carried out by micro-CT analysis using SkyScan 1272 micro-CT device (Bruker micro-CT, Kontich, Belgium). The scanning parameters were as follows: pixel size 3 μm; source voltage 60 kV; source current 166 μA; rotation step 0.2°; rotation 180°; frame averaging 4; and scanning time approximately 2 h for each sample (n = 4). The flat-field correction was updated prior to each acquisition. The reconstruction of cross-section images was carried out from the projection images by NRecon software (Bruker micro-CT, Kontich, Belgium) using a modified Feldkamp algorithm. Three volumes of interest [VOI] (3 mm × 3 mm × sample thickness) were analyzed in each sample. Image-processing and analyses were performed by CTAn (Bruker micro-CT, Kontich, Belgium) and optimized using Test Image Generator software [22]. The porosity (*Po*) was calculated as: *Po* = (volume of open + closed pores)/VOI volume. The pore size was evaluated via a “structure separation” parameter calculated in 3D using a sphere-fitting algorithm [23,24], and the object surface density (OSD) was calculated as OSD = object surface/VOI volume.

#### Structural stability

The structural stability was analyzed in terms of mass loss and swelling ratio. Collagen sponges (8 mm × 8 mm × 4 mm) were immersed in phosphate buffered saline (PBS, pH 7.4, Sigma-Aldrich) in 5% CO_2_ atmosphere at 37°C. The volume of the medium was kept at a ratio of 30 mg/15 mL (sample weight/PBS volume). The mass loss (D) was calculated as D = 100 × (*W_o_* – *W_t_*)/*W_o_* [%], where the initial dried weight of the sample (n = 6) is *W_o_* and the dried weight following degradation is *W_t_*. The dried weight of the samples was measured following lyophilization. The swelling ratio (*E_sw_*) was calculated as: *E_sw_* = 100 × (*W_sw_* – *W_t_*)/*W_o_* [%], where the initial weight is *W_o_*, the dried weight of the sample following degradation is *W_t_*, and the weight of the swollen sample is *W_sw_* (n = 6). The weight of the swollen samples was measured after the removal of the sample from the medium and after a 1-minute delay and the removal of any excessive medium surrounding the sample.

#### Infrared spectrometry

The secondary structure of collagen sponges was evaluated by attenuated total reflection Fourier transform infrared (ATR-FTIR) spectrometry using a Protégé 460 E.S.P. infrared spectrometer (Thermo Nicolet Instruments, Madison, WI, USA) equipped with an ATR device (GladiATR, PIKE Technologies, Madison, WI, USA). All the spectra were recorded in absorption mode, in the range of 4000–400 cm^-1^ with a resolution of 4 cm^-1^ and 128 scans. The integral absorbance (areas of the bands) was determined using deconvolution procedure (OMNIC 7 software, Thermo Nicolet Technologies).

### Vancomycin release tests

Pieces of the collagen sponges sized 10 mm × 20 mm were studied. Four pieces were obtained from two different sponges (two from the edges and two from the central parts) in order to test the variability of the vancomycin content between the sponges and the homogeneity of the vancomycin content within the sponges. The weight of tested samples was 43.0 ± 3.9 mg. The expected amount of vancomycin in the pieces of tested sponges according to the 4:5 collagen: vancomycin ratio was 19.1 ± 1.8 mg. Each tested piece was submerged in 10 mL of 37°C PBS (Sigma-Aldrich, St. Louis, Missouri, USA) in a test tube and gently rocked on a laboratory rocker placed in an incubator at 37°C. The pieces of sponges were transferred to a newly tempered 10 mL of PBS after 30, 60, 120, 180, 240, 330, and 480 min and 24 h. The final samples were obtained 7 days following the commencement of the experiment. The samples of PBS with released vancomycin were subsequently analyzed by a nephelometric immunochemical assay (Beckman Coulter, Indianapolis, IN, USA) for the concentration of vancomycin.

### Disk diffusion test

Six discs of 6 mm in diameter were cut from sponges, three from different sponges and additional three from one sponge (corner, edge, and central part). The microbiological response was tested via a standard disk diffusion test with methicillin-resistant *Staphylococcus aureus* (MRSA), strain CNCTC 6271.

### Rat model of infected wound

Male Wistar rats (n = 30) with a median weight of 280.5 g (interquartile range [IQR] = 272.25–285 g) were anesthetized with isoflurane. An approximately 1-cm long incision was then made on both sides of the back at least 2 cm apart, followed by an injection of a MRSA solution (0.2 mL of 1 × 10^8^ colony-forming unit [CFU]/mL) into a subcutaneous (s.c.) pocket created by a blunt dissection on each side.

With respect to vancomycin group (n = 12), a 10 mm × 10 mm piece of vancomycin-containing collagen sponge was inserted into the pocket on the sponge side and the wound was closed with a non-resorbable monofilament suture. The weight of the pieces applied was 25.2 ± 1.6 mg (mean ± standard deviation [SD]) and the amount of vancomycin in the collagen sponge pieces was 11.2 ± 0.7 mg (mean ± SD). For placebo group (n = 6), a 10 mm × 10 mm piece of placebo-containing collagen sponge was applied. The pocket on the sham surgery side was sutured with no sponge so as to provide for a control.

Along with the active and control arms, additional 12 rats were used for the determination of vancomycin pharmacokinetics. We administered 7.5 mg of vancomycin intraperitoneally to rats accompanied by a similar induction of s.c. infection; we measured the plasma levels in the same way as we did for the active arm.

The animals were then transferred to individual cages with free access to food and water; they were checked daily for weight, body temperature, and behavioral changes. The animals were sacrificed on the 4^th^ post-implantation day. Tissue specimens were then excised from both inoculation sites and examined microbiologically (numbers of MRSA CFU).

### Microbiological evaluation

Tissue samples were imprinted directly on Columbia Agar plates with sheep’s blood (Oxoid Ltd., Basingstoke, UK). The plates were then incubated at 37°C for 24 h. MRSA colonies were counted following incubation.

### Vancomycin and haptoglobin levels in serum

Blood samples (volume, 0.5 mL) for determination of vancomycin and haptoglobin levels were drawn from the jugular vein into a 0.5 mL heparinized syringe under isoflurane anesthesia. Samples were drawn from three animals in vancomycin group 0.5, 1, 2, and 4 h following inoculation on the 1^st^ day to determine vancomycin and haptoglobin levels. Samples were also taken from all animals in placebo group on the 1^st^ day 2 h following inoculation to determine haptoglobin level only. Samples from four animals in vancomycin group and two animals in placebo group were drawn on the 1^st^, 2^nd^, and 3^rd^ day following inoculation. The final sample was drawn intracardially upon termination on day 4 following inoculation. The samples were centrifuged (12,000 rpm, 2 min) and the samples from days 1, 2, and 3 following inoculation were frozen at -20°C until analyzed. Vancomycin concentration in the blood as well as haptoglobin level (rat acute phase marker) were determined using a nephelometric method (Beckman Coulter, Indianapolis, IN, USA).

### Ethical statement

The study was approved by the Animal Care and Use Committee of the First Faculty of Medicine, Charles University (ref. no. MSMT-11255/2015-5) and conducted in accordance with Czech Act No. 246/1992 Coll. as amended on the protection of animals against cruelty according to the EU legislation.

### Statistical analysis

Shapiro–Wilk and Chi-square tests were used for the verification of data normality. Grubbs’ or Dixon’s tests were applied for outlier identification. The variance was checked by the Bartlett’s Levene’s tests. The Kruskal–Wallis test with Bonferroni procedure was used for multiple sample comparisons. The Mann–Whitney U test was applied for two-sample comparisons in the case of violation of normality or homoscedasticity assumptions. In that case, values are expressed as median with IQR. All tests were conducted using Statgraphics Centurion XVII (StatPoint, Warrenton, VA, USA). ANOVA and t-test were used for data expressed as mean ± SD. Fisher’s exact test was applied to data in the contingency tables. The visualization of the microbiological, gentamicin release, and *in vivo* tests was performed using GraphPad Prism version 7.00 for Windows (GraphPad software, La Jolla, CA, USA). Statistical significance was accepted at *p* ≤ 0.05.

## RESULTS

### Microstructure of collagen sponges

**FIGURE 1:**
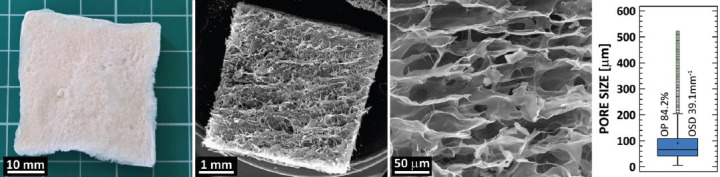
Photograph and scanning electron microscopy micrographs (from left) of the surface and the inner structure of vancomycin-containing collagen sponges. The box-plot shows the pore size, open porosity (OP), and object surface density (OSD) of the prepared samples (n = 4). The median pore size was 66 μm (interquartile range 42–120 μm) and OP was 84.2%. The degree of closed porosity of sponges was very low, i.e., between 2 × 10^-5^ and 4 × 10^-3^%. This value indicates minimal proportion of non-interconnected pores. Finally, OSD was determined at 39.1 mm^-1^.

Figure 1 shows the image and SEM images of collagen sponges. The median pore size was 66 μm (IQR 42–120 μm) and the open porosity (OP) was 84.2% (Figure 1). The degree of closed porosity of sponges was very low, i.e., between 2 × 10^-5^ and 4 × 10**^-3^**%. This value indicates minimal proportion of non-interconnected pores. Finally, the OSD was determined at 39.1 mm^-1^.

### Structural stability of collagen sponges

**FIGURE 2:**
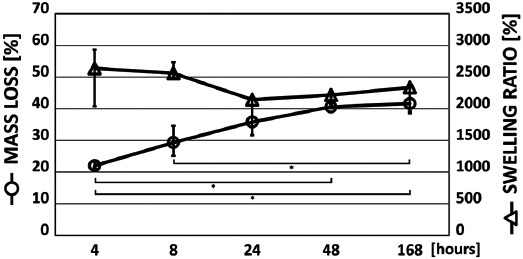
Mass loss and swelling ratio (median, interquartile range) of vancomycin-loaded collagen sponges. The asterisks denote statistically significant differences at the 95% confidence level (Kruskal–Wallis test with Bonferroni procedure). The degradation rate of collagen samples increased rapidly to 22% (median) during the initial 4 h. This initial rapid mass loss may have been the result of the release of vancomycin from the inner structure of the sponges. The mass loss had been almost linear between 4 and 48 h. Subsequently the mass loss did not continue. The maximal mass loss was approximately 41%, indicating stability and gradual degradation of the samples.

The degradation rate of collagen samples increased rapidly to 22% (median) during the initial 4 h (Figure 2). This initial rapid mass loss may have been the result of the release of vancomycin from the inner structure of the sponges. The mass loss had been almost linear between 4 and 48 h. Subsequently the mass loss did not continue. The maximal mass loss was approximately 41%, indicating stability and gradual degradation of the samples.

Vancomycin-loaded collagen samples exhibited considerable swelling with approximately constant swelling ratios during the first 168 h (Figure 2). The constant swelling ability indicates appropriate exchange of body liquids despite the 40% mass loss.

### FTIR analysis of collagen sponges

**FIGURE 3:**
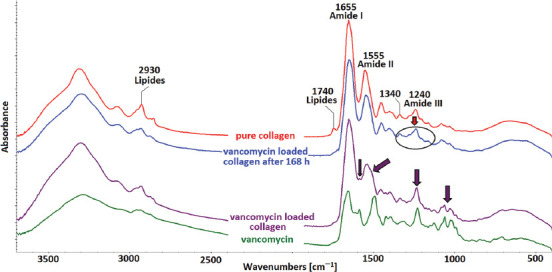
Infrared spectra of vancomycin-loaded collagen materials before and after the degradation tests in phosphate buffered saline. The almost total release of vancomycin was proven. The spectral features of vancomycin disappeared from the spectra of tested collagen sponges after 7 days. The small amount of vancomycin that was not released to the medium was most probably bound to the sponge.

Infrared spectroscopy (FTIR) can be used as an analytical technique to determine changes in the secondary structure of collagen after various processes, such as isolation, crosslinking, denaturation, and sterilization. The FTIR spectra of collagen includes amidic bands such as amide I bands (~1655 cm^-1^) that arise from C=O stretching vibrations coupled with N–H bending vibrations, and also amide II bands (~1555 cm^−1^) and amide III bands (~1240 cm^-1^) that belong to N–H bending vibrations coupled with C–N stretching vibrations (Figure 3). Amide III consists of a band triplet at ~1205, 1240, and 1280 cm^-1^ and, together with another band at 1340 cm^-1^, can be considered as the proof of the existence of a triple helical structure [25,26]. The FTIR spectra of the studied collagen materials containing vancomycin hydrochloride before and after the degradation tests and their comparison with vancomycin hydrochloride and the original collagen material are presented in Figure 3.

The infrared spectra of vancomycin-loaded collagen sponges (violet spectrum) exhibited the fundamental spectrum of pure (without vancomycin) collagen (red spectrum) enriched by the spectral features of vancomycin hydrochloride (violet arrows). The bands at 1740 and 2930 cm^-1^ in the figure 3 are related to the presence of lipids, such as residual impurities that remained within collagen following the isolation procedure [25]. The lipids disappeared from the collagen matter after the immersion in PBS buffer during degradation tests.

The post-degradation spectrum of vancomycin-loaded collagen (blue) revealed disappearance of the vancomycin hydrochloride-related bands, proving that almost all vancomycin was released from collagen sponges within 168 hours. The material preserved its secondary collagen structure beyond 168 h of degradation in PBS medium, as proven by the quartet – amide III (~1205, 1240, and 1280) together with the 1340 band (black ellipse).

### Vancomycin release tests

**FIGURE 4:**
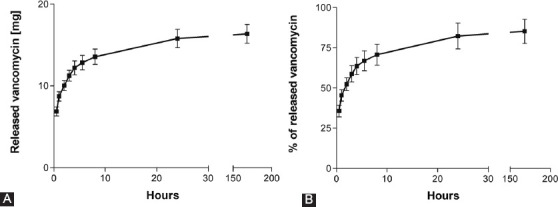
Vancomycin dissolution into phosphate buffered saline. (A) Absolute amount in mg, (B) relative to the expected amount according to the gravimetric assumption. The total amount of vancomycin released from sponges after 7 days was 16.3 ± 2.3 mg, corresponding to 85.25% ± 7.4% of the total vancomycin content. One of the tested sponges was found to have released 79.6% ± 0.9% and another 90.9% ± 5.9% of the expected vancomycin content. Most of vancomycin was released during the first 8 h.

The vancomycin release profile and the percentage of vancomycin released from sponges over time are presented in Figure 4. The total amount of vancomycin released from sponges after 7 days was 16.3 ± 2.3 mg, corresponding to 85.25% ± 7.4% of the total vancomycin content. One of the tested sponges was found to have released 79.6% ± 0.9% and another 90.9% ± 5.9% of the expected vancomycin content. Most of vancomycin was released during the first 8 h (Figure 4).

The almost total release of vancomycin was further proven using infrared spectrometry, which revealed that the spectral features of vancomycin disappeared from the spectra of tested collagen sponges after 7 days (Figure 3). The small amount of vancomycin that was not released to the medium was most probably bound to the sponge.

### Disk diffusion test

**FIGURE 5:**
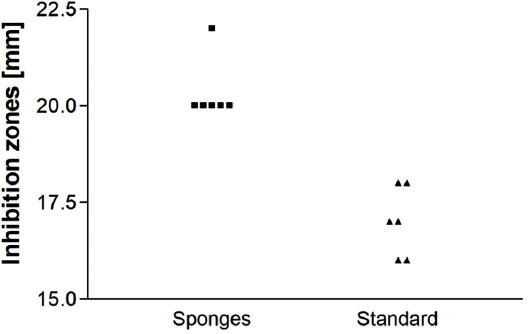
Disk diffusion test of vancomycin-containing collagen sponges and standard susceptibility testing of vancomycin discs. The mean ± standard error of the mean (SEM) of the inhibition zones was 20.3 ± 0.33 and 17.0 ± 0.37 for the sponges and the microbiological standard discs, respectively (p < 0.0001). The tested collagen sponge discs exhibited broad inhibition zones that were even larger than those of standard discs used for testing antibiotic susceptibility.

The tested collagen sponge discs exhibited broad inhibition zones that were even larger than those of standard discs used for testing antibiotic susceptibility (Figure 5). The mean ± standard error of the mean (SEM) of the inhibition zones was 20.3 ± 0.33 and 17.0 ± 0.37 for the sponges and the microbiological standard discs, respectively (*p* < 0.0001).

### Rat model of infected wound

**FIGURE 6:**
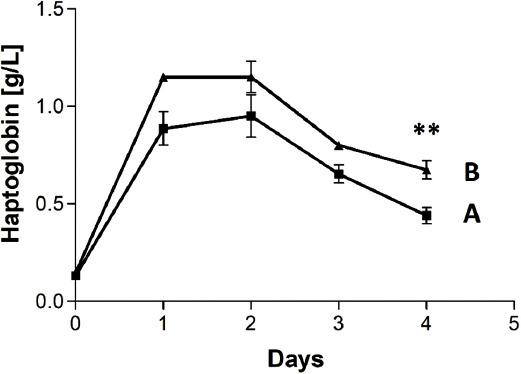
Haptoglobin mean ± standard deviation (SD) plasma levels for vancomycin- (A) and placebo- (B) containing sponge groups. Haptoglobin levels increased in both groups from baseline levels and were higher in placebo group throughout the duration of the study. The difference reached statistical significance on day 4 (p = 0.0034). The haptoglobin area under the curve was 2.78 and 3.51 g*day/L for vancomycin and placebo groups, respectively. **p < 0.01.

No significant changes were determined in terms of the body weight (BW), behavior, or body temperature for either of the groups, although the increase in the median (IQR) weight of animals in vancomycin group was greater on day 4, i.e., 18 g (14.75–20.25) vs. 4 g (-5.75–13.75). Haptoglobin levels increased in both groups from baseline levels and were higher in placebo group throughout the duration of the study. The difference reached statistical significance on day 4 (*p* = 0.0034; Figure 6). The haptoglobin area under the curve (AUC) was 2.78 and 3.51 g*day/L for vancomycin and placebo groups, respectively.

### Pharmacokinetic analysis of intraperitoneal (i.p.) vancomycin

**FIGURE 7:**
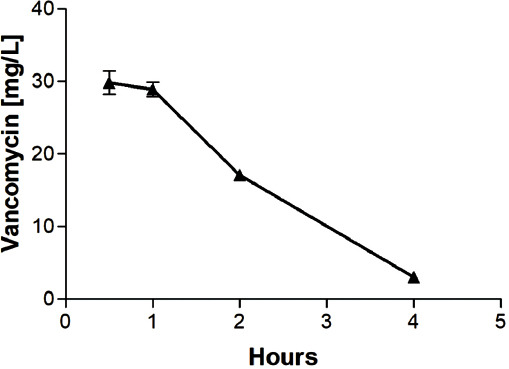
Mean ± standard deviation (SD) vancomycin levels in rats administered with 7.5 mg of vancomycin intraperitoneally. Almost all vancomycin was eliminated during the first 4 h following administration. Vancomycin was detected in one animal only after 24 h (0.2 mg/L). The area under the curve calculated according to the obtained levels was 87.71 mg*h/L.

The mean vancomycin levels following i.p. administration of 7.5 mg of the antibiotic are shown in Figure 7. Almost all vancomycin was eliminated during the first 4 h following administration. Vancomycin was detected in one animal only after 24 h (0.2 mg/L). The AUC calculated according to the obtained levels was 87.71 mg*h/L. Following the administration of 11.2 mg (the same amount that was administered via the sponges), the AUC was expected to be 130.9 mg*h/L.

### Pharmacokinetic analysis of vancomycin released from sponges

**FIGURE 8:**
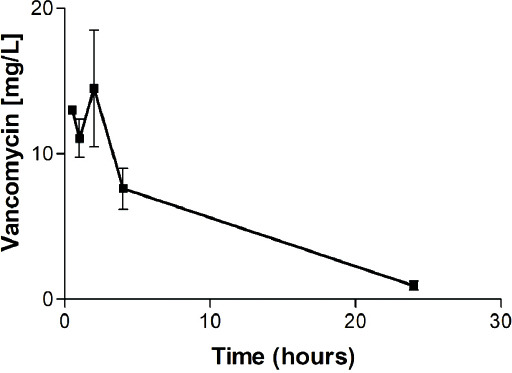
Mean ± standard deviation (SD) vancomycin plasma levels in rats treated with vancomycin-containing sponges. The release of vancomycin was significantly prolonged compared to that following intraperitoneal administration. Vancomycin was still measurable in all four samples 24 h following implantation (0.93 ± 0.6 mg/L) and subsequently decreased to below detectable limits. The area under the curve (AUC) calculated according to the determined levels was 115.5 mg*h/L, i.e., 88.2% of the AUC calculated for the same dose by the intraperitoneal pharmacokinetic analysis described above. We assume that the rest of vancomycin was bound to those sponges that were not degraded during the 4-day study period.

The mean vancomycin plasma levels following s.c. administration via the sponges are presented in Figure 8. The release of vancomycin was significantly prolonged compared to that following i.p. administration. Vancomycin was still measurable in all four samples 24 h following implantation (0.93 ± 0.6 mg/L) and subsequently decreased to below detectable limits (Figure 8). The AUC calculated according to the determined levels was 115.5 mg*h/L, i.e., 88.2% of the AUC calculated for the same dose by the i.p. pharmacokinetic analysis described above. We assume that the rest of vancomycin was bound to those sponges that were not degraded during the 4-day study period.

### Microbiological examination of infected wound biopsies

Table 1 shows a comparison of the numbers of CFU cultivated from the two inoculation sites (with and without sponges) in the animals from vancomycin and placebo groups.

**TABLE 1:**
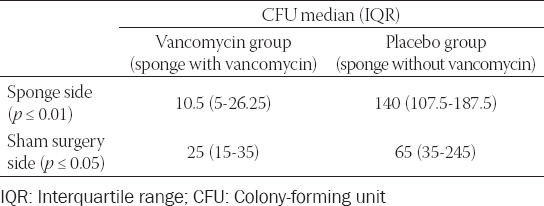
Numbers of CFU cultivated from biopsies taken from the inoculation sites

Even though differences between the sham surgery and the sponge sides in vancomycin and placebo groups were not statistically significant, the numbers of CFU were lower in the treated side in vancomycin group and in the side without sponges in placebo group.

## DISCUSSION

This study presents the preparation of a novel carbodiimide cross-linked freshwater fish collagen sponge containing vancomycin via double lyophilization method and *in vitro* and *in vivo* testing of the resulting sponge. Excellent stability was attained via crosslinking. Following an initial rapid mass loss caused by the release of the antibiotic, the sponges remained compact and stable until at least day 7 in a simulated body environment. To the best of our knowledge, this is the first time freshwater fish collagen is combined with vancomycin to form a hemostatic sponge, which also prevents wound infection due to the Gram-positive bacteria most commonly found on the skin. In addition, it may prevent wound infection in the case of surgery in MRSA contaminated area.

Vancomycin is a partly time-dependent antibiotic and the value of AUC_24_/minimum inhibitory concentration (MIC) ratio as the primary PK/PD parameter should exceed 400 to ensure clinical efficacy in the case of MRSA infections [27]. Thus, vancomycin for systemic infections should ideally be administered repeatedly or via continuous infusion with a loading dose sufficient for rapid and effective exposure [28,29]. Regarding the treatment of local surgical site infections, the ideal PK/PD characteristics can be achieved by slowing down the release of vancomycin from a collagen dressing applied directly to the wound. With the retardation of vancomycin release from the sponge, the drug levels should still be sufficiently high over a reasonable period of time to achieve an AUC_24_/MIC ratio of over 400. Since drug absorption depends not only on the drug concentration at the site of action but also on local vascularization [30], the degree of protection may be higher in low vascularized wounds. This may even be advantageous since such wounds are otherwise difficult to treat. Moreover, in the case of surgical wound infection prophylaxis this may provide the so-called “protected coagulum”, which would normally be attainable only via the application of high doses of intravenous (i.v.) vancomycin with related potential adverse effects. In addition, drug distribution variability may play a role in peripheral infective focus treatment or prophylaxis via systemic drug administration. Therefore, the administration of the drug must be carefully timed to achieve effective levels in the tissue during surgery. Since the antibiotic concentration in a surgical coagulum is approximately 10 times lower than in plasma, even with the proper timing of the administration of the antibiotic [31], the plasma levels of i.v. vancomycin required for effective surgical wound protection would be toxic.

We prepared sponges that retarded the release of vancomycin for at least 8 hours, according to the results of the *in vitro* tests (Figure 4). We also determined significantly different vancomycin plasma profiles following i.p. administration and s.c. administration via the tested sponges in rats (Figures 7 and 8). Although the bioavailability was similar (88.2% of the AUC obtained via i.p. administration), the release of vancomycin to the plasma compartment was markedly slower in the case of administration via the sponges. Therefore, the vancomycin levels in the wound had to be much higher than those achievable by i.v. administration. This allows effective local exposure for the treatment of wound infections by vancomycin as a time-dependent antibiotic. Interestingly, this is in contrast to our former results concerning gentamicin-releasing sponges, where the release profiles were the same for both intramuscular (i.m.) administration and administration via s.c. implanted sponges [3].

The microbiological results of disk diffusion tests revealed effective release of vancomycin as well as effective treatment of wounds infected by MRSA, i.e. the CFU numbers in vancomycin-treated rats were significantly lower than in control group (Table 1). Moreover, the levels of haptoglobin, which is an acute phase marker in rats, were lower in the treated group (the AUC was 21% lower) during the whole duration of the experiment.

The wound care procedure for postoperative wounds in the clinical setting, consisting of daily replacement of wound dressing and the application of vancomycin by sponges that allow slow release of the drug, would be ideal in this case in terms of maintaining effective exposure to the drug over a sufficiently long time period. We prepared sponges that release almost all vancomycin content over 8 hours *in vitro* but continue to release vancomycin after 24 hours *in vivo*. Our sponges would, therefore, release most of the drug before a replacement of the dressing, and the release would be slow enough to prolong the local exposure to the levels above the MIC for at least most of the wound dressing replacement period. Nevertheless, it is important to consider that local vancomycin levels may differ according to vascularization in the treated tissue and may, therefore, vary according to the type of wound (e.g., crural ulcers vs. sternotomy).

By exposing collagen sponges to PBS we showed that approximately 60% of the material remained after 168 hours; moreover, using infrared spectroscopy we confirmed that the material retained its collagen secondary structure. This collagen sponge provided a high degree of OP (over 80%), which plays an important role in terms of fluid mass transport (i.e. nutrients and oxygen), removal of waste and, potentially, cell penetration through the sponge. Suchý et al. [32] showed that collagen scaffold hydration does not affect OP, thus ensuring stable permeability over time. Therefore, following elimination of infection during the first few days, the sponge can remain in the wound as a biocompatible scaffold for the enhancement of granulation and epithelization.

We found no significant difference in terms of CFU count between the sites with and without sponge administration. This may be the result of local vancomycin diffusion to the MRSA inoculation sites not protected by the sponges, due to the small size of the rats and the fact that the treated and control sites were not located far enough from each other. A second possible explanation includes the systemic effect of vancomycin absorbed from the administration site. The situation would, clearly, be very different in larger animals and humans, considering that the distribution volume would be much larger and the dose administered would be much smaller in relation to the BW. Nevertheless, in the case of large wounds involving the use of several sponges, it should be borne in mind that the drug from the sponges is finally absorbed into the systemic circulation and systemic adverse effects may occur. This is of particular importance with respect to renal patients since the half-life of vancomycin is significantly extended in cases of renal failure [33].

## CONCLUSION

This study presents an effective antimicrobial hemostatic wound dressing that is able to serve as a ready-to-use device and that will assist surgeons with respect to both surgical wound infection prophylaxis and the treatment of local infection. We employed freshwater fish collagen, which is less immunogenic than mammal collagen, and increased the stability of the collagen via crosslinking with carbodiimide. The added antibiotic is vancomycin which is not commonly used in combination with hemostatic wound dressing and may prevent surgical wound infection caused by Gram-positive bacteria commonly found on the skin. The other possible use is to prevent infection during surgery involving MRSA contaminated area. The sponge remained stable at 37°C for at least 7 days and released the vancomycin content gradually for several hours following implantation. The efficacy of the treatment of infected wounds was proven in a rat model, by a decrease in the number of MRSA CFUs in the infected site following the local administration of vancomycin-releasing sponge.
